# RETRACTION: Evaluating the Impact of Ultrasound‐Guided Subsheath versus Extrasheath Sciatic Nerve Block on Postoperative Wound Pain in Tibial and Foot Surgeries: A Systematic Review and Meta‐Analysis

**DOI:** 10.1111/iwj.70519

**Published:** 2025-04-21

## Abstract

**RETRACTION:** YuD. , WangX. , JiangL. , WuY. , HanS. , and LiJ. , “Evaluating the Impact of Ultrasound‐Guided Subsheath versus Extrasheath Sciatic Nerve Block on Postoperative Wound Pain in Tibial and Foot Surgeries: A Systematic Review and Meta‐Analysis,” International Wound Journal 21, no. 4 (2024): e14640, 10.1111/iwj.14640.38155428 PMC10961860

The above article, published online on 28 December 2023, in Wiley Online Library (http://onlinelibrary.wiley.com/), has been retracted by agreement between the journal Editor in Chief, Professor Keith Harding; and John Wiley & Sons Ltd. Following an investigation by the publisher, all parties have concluded that this article was accepted solely on the basis of a compromised peer review process. The editors have therefore decided to retract the article. The authors did not respond to our notice regarding the retraction.

**RETRACTION:** D. Yu , X. Wang , L. Jiang , Y. Wu , S. Han , and J. Li , “Evaluating the Impact of Ultrasound‐Guided Subsheath versus Extrasheath Sciatic Nerve Block on Postoperative Wound Pain in Tibial and Foot Surgeries: A Systematic Review and Meta‐Analysis,” International Wound Journal 21, no. 4 (2024): e14640, 10.1111/iwj.14640.38155428 PMC10961860

The above article, published online on 28 December 2023, in Wiley Online Library (http://onlinelibrary.wiley.com/), has been retracted by agreement between the journal Editor in Chief, Professor Keith Harding; and John Wiley & Sons Ltd. Following an investigation by the publisher, all parties have concluded that this article was accepted solely on the basis of a compromised peer review process. The editors have therefore decided to retract the article. The authors did not respond to our notice regarding the retraction.

